# Spatial multi-criteria decision analysis to predict suitability for African swine fever endemicity in Africa

**DOI:** 10.1186/1746-6148-10-9

**Published:** 2014-01-09

**Authors:** William A de Glanville, Laurence Vial, Solenne Costard, Barbara Wieland, Dirk U Pfeiffer

**Affiliations:** 1 The Royal Veterinary College, Hawkshead Lane, Hatfield, Hertfordshire AL9 7TA, UK; 2 Centre International de Recherche Agronomique pour le Developpement, Montpellier, France; 3 Current address: Centre for Immunity, Infection and Evolution (CIIE), Ashworth Laboratories, University of Edinburgh, Edinburgh, UK; 4 Current address: EpiX Analytics, 1643 Spruce Street, Boulder, CO 80302, USA

**Keywords:** African swine fever, Knowledge-driven risk mapping, Multi-criteria decision analysis

## Abstract

**Background:**

African swine fever (ASF) is endemic in several countries of Africa and may pose a risk to all pig producing areas on the continent. Official ASF reporting is often rare and there remains limited awareness of the continent-wide distribution of the disease.

In the absence of accurate ASF outbreak data and few quantitative studies on the epidemiology of the disease in Africa, we used spatial multi-criteria decision analysis (MCDA) to derive predictions of the continental distribution of suitability for ASF persistence in domestic pig populations as part of sylvatic or domestic transmission cycles. In order to incorporate the uncertainty in the relative importance of different criteria in defining suitability, we modelled decisions within the MCDA framework using a stochastic approach. The predictive performance of suitability estimates was assessed via a partial ROC analysis using ASF outbreak data reported to the OIE since 2005.

**Results:**

Outputs from the spatial MCDA indicate that large areas of sub-Saharan Africa may be suitable for ASF persistence as part of either domestic or sylvatic transmission cycles. Areas with high suitability for pig to pig transmission (‘domestic cycles’) were estimated to occur throughout sub-Saharan Africa, whilst areas with high suitability for introduction from wildlife reservoirs (‘sylvatic cycles’) were found predominantly in East, Central and Southern Africa. Based on average AUC ratios from the partial ROC analysis, the predictive ability of suitability estimates for domestic cycles alone was considerably higher than suitability estimates for sylvatic cycles alone, or domestic and sylvatic cycles in combination.

**Conclusions:**

This study provides the first standardised estimates of the distribution of suitability for ASF transmission associated with domestic and sylvatic cycles in Africa. We provide further evidence for the utility of knowledge-driven risk mapping in animal health, particularly in data-sparse environments.

## Background

African swine fever (ASF) is a severe and highly contagious viral infection of domestic pigs. Outbreaks have been reported from almost all the countries of Africa that occur on or below the equator, as well as several countries of West Africa and the islands of Madagascar and Cape Verde [1,2]. The disease can impose severe limitations on pig production in endemic countries, as well as in adjoining areas, which may be at high risk of virus introduction [3,4]. The ASF virus (ASFV) has repeatedly spread outside Africa via the movement of pigs or pig products and continues to pose a considerable threat to pig production in the wider world [1].

Conditions leading to ASF endemicity in domestic pigs in Africa are not fully understood but are likely to vary on a regional basis. In parts of eastern and southern Africa, for example, cycles involving wild suids, such as warthogs (*Phacochoerus africanus*) and soft tick vectors of the *Ornithodoros moubata* complex provide a reservoir of ASFV for domestic pigs [5]. Such ‘sylvatic’ cycles may allow the disease to persist in pig populations, even in areas with low pig population density. In parts of West Africa, on the other hand, sylvatic cycles have been infrequently described and endemicity is more likely to be the result of sustained virus transmission within high density and highly connected pig populations [2,6-8]. Factors contributing to the transmission of ASF in these ‘domestic’ pig cycles may include the use of free-ranging husbandry methods, the movement of infected pigs between farms and to markets, and the feeding of contaminated domestic waste to pigs [1]. African swine fever endemicity in domestic pig populations in Africa may therefore be the result of cycles involving repeated virus introduction from wild suid reservoirs, sustained virus spread within domestic pig populations, or a combination of both.

Risk maps can be used to provide a visual representation of the spatial distribution of the risk of an event, and have been advocated as having a role in the development of targeted disease surveillance and control activities [9,10]. Disease risk maps are traditionally developed as an extension of a statistical modelling process in which geographically explicit predictor variables are used to estimate the probability of disease occurrence [11-13]. Such ‘data-driven’ methods are limited to those areas in which surveillance activities have effective coverage, or in which epidemiological studies can provide adequate data to describe the distribution of a disease. In areas where these data are unavailable, as is often the case for animal diseases in developing countries, a more pragmatic approach to risk mapping has been proposed [14,15]. ‘Knowledge-driven’ risk mapping is one such approach that uses literature based-evidence or expert opinion, rather than a empirical exploration of available data, to describe the relative importance of risk factors for a disease [16]. In data sparse environments, this evidence can be integrated into a formal decision making process to predict the suitability of a geographic area for disease occurrence based on the presence of the identified risk factors.

The likelihood of infectious disease occurrence is typically influenced by multiple interacting factors. Knowledge-driven disease risk mapping should therefore be performed as part of a formal and systematic evaluation framework that takes into account the relative contribution each factor makes to the overall estimation of suitability. Multi-criteria decision analysis (MCDA), a methodology that allows the analysis of complex decision problems involving conflicting criteria, has previously been used within a geographic information system (GIS) for this purpose [14,15,17,18]. Broadly, MCDA allows the prioritisation of the criteria that influence a decision, and provides a framework by which users can reach a decision that reflects these priorities. In the context of risk mapping, ‘criteria’ are risk factors for an undesirable event, such as disease occurrence, while the ‘decision’ relates to the estimation of suitability of an area for the event, and therefore the relative likelihood that it can occur.

In the absence of available (or reliable) disease data to adequately describe the distribution of ASF in domestic pigs in Africa, we used a spatial MCDA to predict suitability for repeated introduction of the virus into pig populations from sylvatic reservoirs and suitability for sustained spread within domestic pig populations. African swine fever has a complex epidemiology with multiple pathways for introduction and spread [1]. There remains considerable uncertainty about the relative importance of these different transmission routes, and the risk factors that influence them, particularly in Africa where few quantitative epidemiological studies have been performed. In order to incorporate some of this uncertainty into the MCDA procedure, we used a probabilistic framework that describes the contribution each risk factor makes to suitability of an area for ASF persistence in domestic pig populations via sylvatic or domestic transmission cycles.

## Methods

### Multi-criteria decision analysis (MCDA)

The MCDA procedure used in this study involved the following general steps (modified from Store and Kangas [19]):

1. *Assessment of the suitability structure*: identifying risk factors for ASF transmission and determining their importance in relation to the objective(s).

2. *Producing spatial layers*: raw data acquisition and transformation to the appropriate GIS layers.

3. *Cartographic modelling*: combining risk factor layers based on the suitability structures defined.

4. *Validation*: comparison of suitability predictions with available disease data.

### Assessment of the suitability structure

#### Probable risk factors for ASF transmission in Africa

On the basis of a literature review, risk factors were identified that are expected to influence the suitability of an area for repeated transmission of ASF into domestic pig populations from sylvatic reservoirs (the ‘sylvatic’ cycle) *(objective 1)* or suitability for sustained transmission of ASF within domestic pig populations (the ‘domestic’ cycle) (*objective 2*). Repeat transmission via either cycle could be expected to lead to ASF persistence and endemicity. Risk factors for ASFV transmission, and the availability of evidence to support their role in the epidemiology of the disease, have been extensively reviewed in a number of recent papers [1,2,5,7,20], and we thus provide only a summary of the literature.

Warthogs (*Phacochoerus africanus)* have long been recognised as a major reservoir of ASFV for domestic pigs in Africa and outbreaks are frequently reported when pigs are reared in areas in which warthogs are common [5]. The transmission of ASFV from infected warthogs to domestic pigs typically occurs via *Ornithodoros* spp. tick vectors [21], although transmission may also occur as a result of contact with contaminated wild suid carcasses [22]. Free-living bushpigs (*Potamochoerus porcus* and *P. larvatus*) and giant forest hogs (*Hylochoerus meinertzhageni*) have also been found to be infected with ASFV [5] and may therefore act as a reservoir in areas in which they occur. The epidemiological significance of bushpigs or giant forest hogs, and the extent to which these species interact with *Ornithodoros* spp. ticks in the transmission of ASFV to domestic pigs, has not been fully determined, but is generally considered to be small in comparison to warthogs [5,20].

As well as parasitising wild suids, *Ornithodoros* spp. inhabit cracks and crevices in pig pens and people’s homes, where they may feed on domestic pigs [23]. Some *Ornithodoros* spp. ticks have been shown to be capable of transovarial, transtadial and sexual transmission of ASFV [24], and can survive for long periods without feeding [25]. These tick vectors may therefore contribute to the persistence of ASF within domestic pig populations, even in the absence of wild suid reservoirs and, occasionally, in the long term absence of viraemic domestic pig hosts [26]. *Ornithodoros* spp., which occur widely in sub-Saharan Africa [23,27-29], are thought to contribute to disease endemicity in Malawi [30], but little evidence has been found for a role for soft tick vectors in domestic (or sylvatic) cycles in West Africa [31], and few studies have been conducted in the rest of sub-Saharan Africa.

The role of pig population density has not, to our knowledge, been determined for ASF in Africa, although it was found to be a predictor of disease risk during recent outbreaks in Russia [32]. High pig or pig farm density has been shown to be associated with infection risk for a variety of viral pig diseases [33,34] and the risk of introduction of infection from wildlife has been related to higher domestic animal density more generally [35].

Infected pigs can shed ASFV for several weeks, hence pig movement between farms, to markets and to slaughter is likely to be a major route by which the virus spreads within domestic pig populations [1], although there have been relatively few studies to quantify the importance of these factors in Africa [6,8,36]. A higher density of road networks was found to increase ASF risk in Russia [32], further highlighting the probable importance of trade on disease transmission as part of the domestic cycle.

Given the available information, we identified the following risk factors as potentially contributing to the suitability of an area for objective 1 (‘sylvatic cycles’): occurrence of warthogs; occurrence of bushpigs; occurrence of giant forest hogs; occurrence of *Ornithodoros* tick spp.; pig population density. The following factors were considered important in describing the suitability of an area for objective 2 (‘domestic cycles’): proximity to major market centres, acting as a proxy for trade in pigs and their products; pig population density; and the occurrence of *Ornithodoros* tick spp.

### Suitability structure

**Figure 1:**
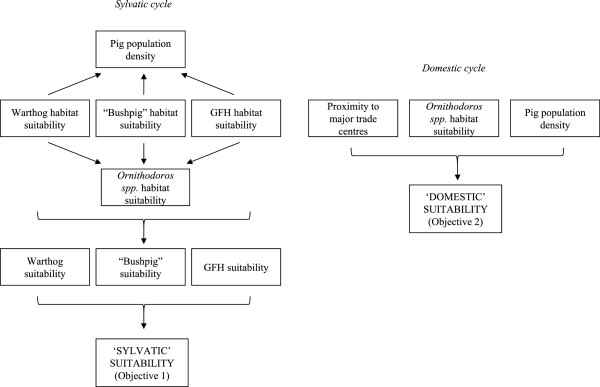
Framework for the MCDA procedure.

Warthogs, bushpigs and giant forest hogs are likely to have variable importance in the epidemiology of ASF in Africa, with each interacting differently with *Ornithodoros* spp. and pig population density in the transmission of the disease to domestic pigs [5]. The suitability of an area for the introduction of ASF from wildlife reservoirs (*objective 1*) was therefore considered using the hierarchical approach shown in Figure 1. For this, the likelihood of introduction of ASF from each species was assessed separately based on the interaction between wild suid habitat suitability (as a proxy for probability that the wild suid species occurs in an area), *Ornithodoros* spp. habitat suitability (as a proxy for the probability tick vectors occur in an area) and pig population density. The overall suitability could then be considered as the weighted average of the individual suitabilities for each wild suid species, with weights assigned based on the perceived importance of each species in the transmission of ASF to domestic pigs. The suitability of an area for sustained spread within domestic pig populations considered the interaction between pig population density, *Ornithodoros* spp. habitat suitability (as a proxy for the probability tick vectors occur in an area) and proximity to major market centres.

As well as these ‘suitability’ criteria (i.e. risk factors), possible constraints on the suitability of an area for sustained transmission of ASFV through sylvatic or domestic cycles were identified. For *objective 1*, constraints on the suitability of an area for the introduction of ASF from each wild suid species were considered to be those areas in which warthogs, bushpigs or giant forest hogs were presumed to be entirely absent (i.e. outside the species’ usual range). For both objectives, areas of Africa in which domestic pigs are absent were considered to constrain suitability for sustained ASF transmission via either cycle.

### Estimating criteria weights

#### Comparing risk factors

A pairwise comparison approach [37] was used to define the relative importance of each of the identified suitability criteria (risk factors) in relation to objective 1 or 2. Each criterion was compared with each of the other criteria for each objective, and assigned a score using the preference statements described in Table 1. Hence, risk factor *y* could be considered equally to extremely more (less) important when compared to risk factor *z* in relation to the objective of interest. To approximate the uncertainty that existed in these estimates of relative importance, a Betapert distribution was created for each pairwise comparison using the @Risk add-on for Microsoft Excel (@Risk for Excel, Version 5.5., Palisade Corp., Newfield, NY). The distribution was defined on the basis of the expected minimum, most likely and maximum preference value for each pairwise comparison.

**Table 1:** **Preference statements and values used in pairwise-comparisons (after Saaty**[35]**)**

**Value**	**Description**
1	Equally important
2	Equal to moderately more important
3	Moderately more important
4	Moderately to strongly more important
5	Strongly more important
6	Strong to very strongly more important
7	Very strongly more important
8	Strongly to extremely more important
9	Extremely more important

All pairwise comparisons were conducted by the lead author based on his own subjective assessment of the evidence available through literature review, and were reviewed with the co-authors who have all previously contributed peer-reviewed articles on aspects of the epidemiology of ASF in Africa. The final minimum, most likely, and maximum preference statements for each pairwise-comparison were those that were agreed by all authors.

#### Risk factor weights

In order to derive a weight for each suitability criterion that would correspond to its relative importance in relation to each objective, a comparison matrix was built using single values drawn from the distributions of pairwise comparisons. The resulting matrix was reciprocal, so that the pairwise-comparison for risk factor *y* and risk factor *z* was, *a*_*yz*_ = *a*_*yz*_^− 1^ and all of its diagonal elements were unity, so that *a*_*yz*_ = 1 when  *y* = *z*.

The preference values for each factor were summarised to derive a vector of weights by normalising the eigenvector associated with the maximum eigenvalue of the pairwise comparison matrix [38], so that:

$$a_{\mathrm{yz}}=\frac{a_{\mathrm{yz}}}{\sum_{y-1}^{1}a_{\mathrm{yz}}\;}\;\text{for}\;\text{all}\;z=1,2....n.$$

and the specific weights for each factor were given by:

$$w_{y}={\hat{a}}_{\mathrm{yz}}$$

To prevent the generation of comparison matrices that could be considered inconsistent (i.e. in which the magnitude of the preference statement assigned to *y* vs. *x* and *z* vs. *x* was not consistent when comparing *z* vs. *y*), we assessed the overall consistency of each comparison matrix by calculating a ‘consistency index’ (CI) as the eigenvector of the normalised pairwise comparison matrix as:

$$\mathrm{CI}=\frac{\gamma_{\max}-p}{p-1}$$

where *γ*_*max*_ is the largest eigenvalue that can be obtained once we have its associated eigenvector and *p* is the number of columns of the matrix [39]. The resultant matrix specific consistency index was then divided by a randomly generated consistency index to produce a ‘consistency ratio’ (CR). Comparisons were considered to be consistent if the CR was less than 0.1 based on the system defined by Saaty [37]. All comparison matrices in which the consistency ratio was 0.1 or greater were discarded. This process was repeated over multiple iterations, with comparison matrices populated by values drawn by Monte Carlo sampling from the Betapert distribution representing each pairwise-comparison, until a large number of consistent matrices and weights (at least 3000) were generated for each risk factor for each of the two objectives. The output was considered to represent the distribution of possible weights for each factor under consideration, given the authors’ uncertainty in the factor’s importance in relation to each objective.

The process was automated using the VBA programming language (Microsoft Corp) and implemented in Microsoft Excel (2010).

### Producing spatial layers

Raster maps for the suitability criteria and constraints identified through the literature review were sourced and mapped in ArcMap 10 (ESRI, Redlands, CA). The source and structure of the spatial data used to represent suitability layers are given in Table 2. In the absence of data of sufficient accuracy to represent areas in which domestic pigs do not occur, a human population density of zero per km^2^ (as defined by Landscan 2008, High Resolution Global Population Data Set, UT-Battelle, LLC, operator of Oak Ridge National Laboratory, US) was considered to provide the best evidence of areas in which domestic pigs cannot occur. This constraint layer and that representing the predicted home ranges of warthogs, the two bushpig species and giant forest hogs (as sourced from the African Mammals Databank (AMD) [40]), were converted into Boolean functions of ‘suitable’ and ‘unsuitable’ and assigned a value of 1 or 0, respectively.

**Table 2:** Data and standardisation approaches to derive suitability layers for the MCDA procedure

**Suitability criterion**	**Source**	**Type**	**Standardisation**		**Shape**
			***x***_***jmin***_	***x***_***jmax***_	
Warthog habitat suitability	African mammals databank (http://www.gisbau.uniroma1.it/amd/)	Continuous probability surface (habitat suitability)	1	RiskUniform(35,50)^1^	Linear decreasing^2^
Bushpig (*P. porcus*) habitat suitability	African mammals databank	Continuous probability surface (habitat suitability)	1	RiskUniform(10,37)^1^	Linear decreasing^2^
Bushpig (*P. larvatus*) habitat suitability	African mammals databank	Continuous probability surface (habitat suitability)	1	RiskUniform(11,26)^1^	Linear decreasing^2^
Giant forest hog habitat suitability	African mammals databank	Continuous probability surface (habitat suitability)	1	RiskUniform(11,40)^1^	Linear decreasing^2^
Pig population density	FAO GLW (http://www.fao.org/ag/AGAInfo/resources/en/glw/GLW_dens.html)	Predicted density surface pigs/km^2^ (adjusted to match FAOSTAT 2005 national totals)	0	RiskUniform(1,14)^1^	Linear increasing
*Ornithodoros* spp. habitat suitability	Unpublished data (Vial and Estrada-Pena)	Continuous probability surface (habitat suitability)	10	100	Linear increasing
Travel time to market centres >20,000	Harvest Choice (http://harvestchoice.org/products/data)	Raster describing travel time (hrs)	0	RiskUniform(15,30)^1^	Linear decreasing

^1^Lower and upper bounds of the distribution defined by the 90^th^ and 99^th^ percentile of values for Africa within area defined by model constraints; ^2^Values describe the Mahalanobis distance from ideal habitat suitability: suitability decreases as distance increases (see [40] for full details).

Continuous values from each raster layer representing the suitability criteria (Table 2) and the Boolean constraints were extracted from each cell of a grid with a cell size 0.8 × 0.8 decimal degrees over the whole of mainland Africa and exported directly into Microsoft Excel, resulting in a dataset consisting of approximately 360,000 individual data points for each of the criteria. Suitability values were standardised to a common monotonic linear scale between 0 and 1 using the maximum score procedure [39], so that for linearly increasing criteria:

x'ij=xijxjmax

And for linearly decreasing criteria:

x'ij=1−xijxjmax

Where *x*_*ij*_ is the raw score for criterion *j* and *x*_*jmax*_  represents the point at which its contribution to predictions of suitability becomes zero: all values above *x*_*jmax*_ were assigned a value of 1 for criteria that increased linearly or 0 for criteria that decreased linearly. Where uncertainty existed in the appropriate value of, *x*_*jmax*_ a uniform distribution was defined using @risk in which maximum and minimum values for *x*_*jmax*_ were described by the 99^th^ and 90^th^ percentile of the distribution of values of each variable within the areas defined as suitable within the constraints for each cycle (Table 2).

### Cartographic modelling

In order to derive an estimate of suitability, *S*, for each sampled point *i* in relation to each objective, the standardised suitability values and the weight for each criterion derived from the pairwise-comparison procedure (*w*) were combined using weighted linear combination (WLC) [39], so that:

$$S=\sum w_{j}x_{\mathrm{ij}}c_{i}$$

where c_*i*_ represents constraint layers coded as 1 or 0 to describe suitability or unsuitability, respectively.

To incorporate the full range of possible combinations of *w* for each criterion, as well as the uncertainty associated with the standardisation of suitability layers, the process was repeated over 3000 iterations, with a specific weight combination randomly selected by Monte Carlo sampling for each iteration. Hence, each sampled point was associated with 3000 possible estimates of suitability (*S*) for each objective based on the linear combination of values of *x*. Three thousand iterations were used to allow the incorporation of the full range of weights (*w*) generated through the iterative pairwise comparison procedure (n = 3000).

The mean value and the 5^th^ and 95^th^ percentiles were extracted from the resulting distributions and exported to ArcMap where point values were converted directly to a continuous raster surface for the visual presentation of results.

To enable formal comparisons of the geographic coverage of areas of predicted suitability for persistence as part of either cycle, we defined a fixed suitability threshold of 0.5 on a 0 to 1 scale [41]. The total land surface area with suitability predictions above this threshold for the whole of Africa and for each of the UN sub-regions of Africa (Northern, Western, Central, Eastern, and Southern Africa) was calculated using the zonal statistics tool in ArcMap and the Lambert Azimuthal Equal Area projection.

### Validation

ASF outbreak report data from 2005 to September 2012 for mainland Africa were downloaded from the World Animal Health organisation WAHID system (OIE, 2012). When geographic co-ordinates for the location of a reported outbreak were not provided but a location name was given, ‘Fuzzy Gazetteer’ (isodp.hof-university.de/fuzzyg) was used to select the most likely co-ordinates for the location of the outbreak.

Validation was performed on the mean suitability estimate for sylvatic cycles, domestic cycles, and a combination of the two. Given the non-independence of predictions for each objective (which were defined using some of the same criteria but with different weightings applied), the combined suitability estimate was derived by combining the mean value for each cycle using an ordered approach where the highest value for either objective was used to define the value at each pixel.

A partial ROC analysis was implemented on the combined suitability estimates and the mean estimates for each cycle using the Partial-ROC tool developed by Barve [42]. Only suitability estimates from countries in which outbreaks had been reported to the OIE were used for validation. The partial ROC is a plot of sensitivity (proportion of correctly predicted disease cases) against the proportion of study area predicted as being suitable for disease occurrence. Hence, the approach does not rely on absence data, which are difficult to generate for ASF for much of Africa, where animal disease surveillance systems are often inadequate. Moreover, the approach allows for the inclusion of a user generated error term (*E*) to account for the amount of error admissible along the true-positives axis (the omission error) [43]. The latitude and longitude describing the geographical location of ASF outbreaks reported to the OIE is typically given at one or two decimal places, and represents the focus of an outbreak that can cover a wide geographic area: the validation dataset can therefore be expected to contain substantial amounts of geo-referencing based error that could contribute to omission error.

The partial ROC output is the ratio of the area under the curve (AUC) of the restricted ROC curve (i.e. the region where the omission error is less than *E*) against the AUC of the restricted null model. Bootstrapping to evaluate the statistical significance of the AUC ratios was performed by resampling 50 % of test points 1000 times from the pool of outbreak data, as described by Peterson et al. [43]. The value of *E* was set at 20 % to represent the likely high levels of geo-referencing error present in the validation set.

## Results

### Multi-criteria decision analysis

#### Criteria weights

##### Objective 1

The pairwise-comparisons used for objective 1 are shown in Table 3 and the resulting weightings for each criterion in Table 4.

**Table 3:** Minimum, most likely and maximum values for the pairwise comparison of criteria for objective 1

Pairwise comparison*	**Min**	**Most likely**	**Max**
Warthog habitat suitability vs. Pig population density	1	5	9
*Ornithodoros.* spp*.* habitat suitability vs. Pig population density	1	3	9
*Ornithodoros.* spp. habitat suitability vs. Warthog habitat suitability	1	1	5
Bushpig habitat suitability vs. Pig population density	1	5	9
*Ornithodoros.* spp*.* habitat suitability vs. Pig population density	1/9	1/5	1/3
*Ornithodoros.* spp. habitat suitability vs. Bushpig habitat suitability	1/9	1/7	1/5
Giant forest hog (GFH) habitat suitability vs. Pig population density	1	5	9
*Ornithodoros.* spp. habitat suitability vs. Pig population density	1/9	1/7	1/5
*Ornithodoros.* spp. habitat suitability vs. GFH habitat suitability	1/9	1/9	1/7
Warthogs vs. Bushpigs	1	4	7
GFHs vs. Bushpigs	1/9	1/5	1/3
GFHs vs. Warthogs	1/9	1/9	1/7

*Minimum, most likely and maximum values defined based of the comparison of the importance of the first criterion in relation to the second for the objective under consideration using the preference statements in Table 1.

**Table 4:** **Distributional estimates of weights for criteria for objective 1 derived from the pairwise comparisons in Table **3**(see text)**

	**Criteria**	**Mean**	**5**^**th **^**percentile**	**95**^**th **^**percentile**
Warthog	Warthog habitat suitability	0.40	0.30	0.48
	Pig population density	0.11	0.076	0.16
	*Ornithodoros.* spp. habitat suitability	0.49	0.41	0.59
Bushpig	Bushpig habitat suitability	0.63	0.54	0.71
	Pig population density	0.29	0.22	0.38
	*Ornithodoros.* spp. habitat suitability	0.077	0.066	0.089
GFH	GFH habitat suitability	0.64	0.57	0.69
	Pig population density	0.30	0.26	0.37
	*Ornithodoros.* spp. habitat suitability	0.060	0.055	0.066
Wild suids	Warthog	0.66	0.57	0.73
	Bushpig	0.27	0.20	0.36
	Giant forest hog	0.067	0.059	0.076

The bushpigs and giant forest hogs show a similar pattern of suitability weightings, with habitat suitability considered to be the most important factor in determining the suitability of an area for repeated introduction of ASF from these species, followed by pig density with relatively minor contributions from tick habitat suitability. Tick habitat suitability was considered to be substantially more important in the suitability of an area for sustained transmission of ASF to domestic pigs from warthogs.

The preference values used to describe the general importance of each wild suid species in acting as a reservoir of ASF for domestic pigs are also shown in Table 3, and the associated criterion weights in Table 4. Warthogs were considered to be the most important reservoir species for ASF for domestic pigs, followed by the bushpigs. Giant forest hogs were considered to be comparatively unimportant.

##### Objective 2

The preference statements from the pairwise estimation of the relative importance of each criterion in relation to objective 2 and the associated weightings are presented in Table 5 and Table 6, respectively. Pig population density was considered to be the most important factor influencing the sustained spread of ASF within domestic pig populations, with proximity to market centres and tick habitat suitability making smaller contributions.

**Table 5:** Minimum, most likely and maximum values for the pairwise comparison of criteria for objective 2

Pairwise comparison*	**Min**	**Most likely**	**Max**
*Ornithodoros.* spp. habitat suitability vs. Pig population density	1/7	1/5	1/3
Proximity to market centres vs. Pig population density	1/5	1/3	1
Proximity to market centres vs. *Ornithodoros.* spp. habitat suitability	3	5	7

*Minimum, most likely and maximum values defined based of the comparison of the importance of the first criterion in relation to the second for the objective under consideration using the preference statements in Table 1.

**Table 6:** **Distributional estimates of weights for criteria for objective 2 derived from the pairwise comparisons in Table **5**(see text)**

**Risk factor**	**Mean**	**5**^**th **^**percentile**	**95**^**th **^**percentile**
Pig population density	0.56	0.49	0.63
Proximity to market centres	0.35	0.28	0.41
*Ornithodoros.* spp. habitat suitability	0.093	0.078	0.11

### Cartographic modelling

#### Objective 1

**Figure 2:**
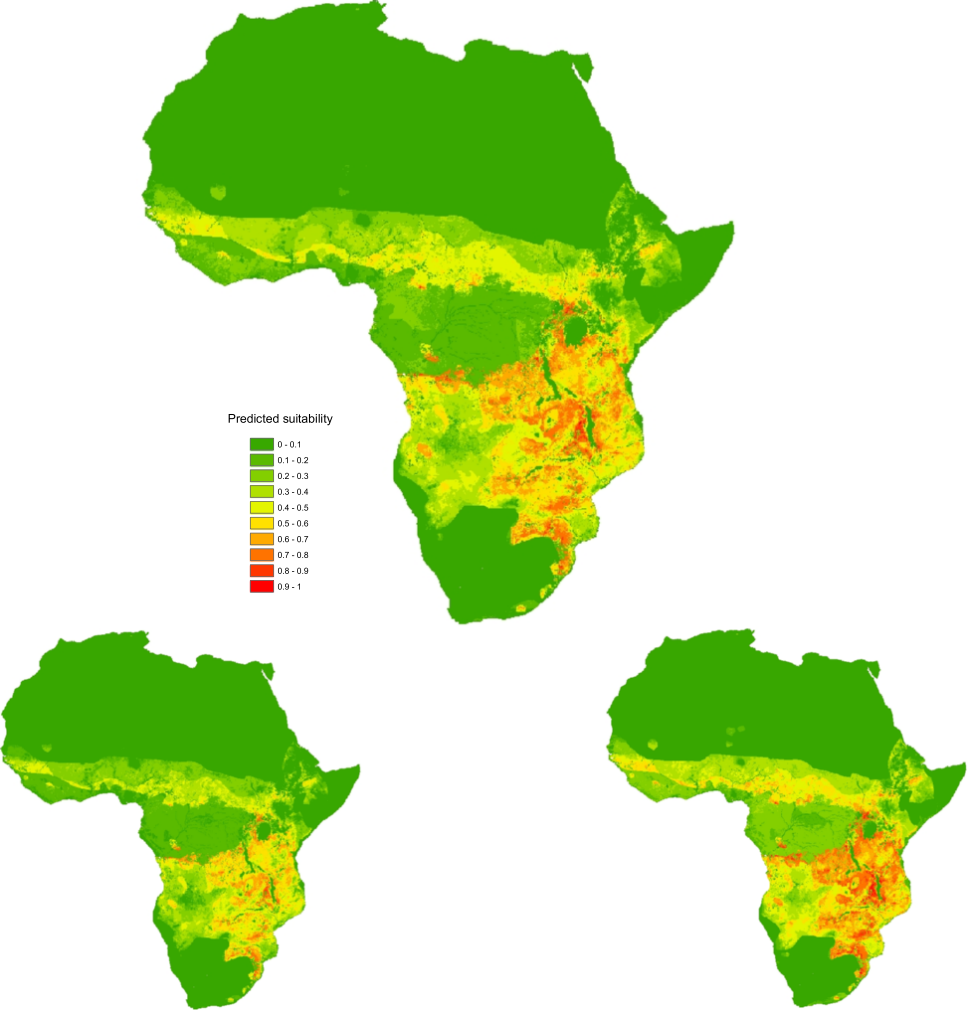
**Suitability for ASF persistence as part of sylvatic cycles (5**^**th **^**percentile (left); average (middle); 95**^**th **^**percentile (right)).**

Areas with the highest estimated suitability for repeated introduction of ASF into domestic pig populations from sylvatic reservoirs were predicted in East and Central sub-Saharan Africa, with sustained sylvatic transmission appearing to be relatively unlikely in West Africa, as well as the vast majority of northern and south-western Africa (Figure 2 and Table 7).

**Table 7:** **Total predicted land area above a 0.5 suitability threshold for the distributional estimates (5**^**th **^**percentile, mean and 95 % percentile) for each transmission cycle in the UN sub-regions of Africa**

	**Region**	**Sub-region land area (Km**^**2**^**)**	**Land area predicted above 0.5 threshold (Km**^**2 **^**(% of total))**		
			**5**^**th **^**percentile**	**Mean**	**95**^**th **^**percentile**
Sylvatic	Western Africa	60 x 10^5^	56 x 10^2^**(0.09)**	81 x 10^3^**(1.3)**	25 x 10^4^**(4.2)**
	Central Africa	66 x 10^5^	61 x 10^4^**(9.3)**	87 x 10^4^**(13.2)**	13 x 10^5^**(20.2)**
	Southern Africa	27 x 10^5^	22 x 10^4^**(8.3)**	31 x 10^4^**(11.5)**	40 x 10^4^**(14.9)**
	Eastern Africa	58 x 10^5^	14 x 10^5^**(23.7)**	20 x 10^5^**(34.9)**	25 x 10^5^**(44.0)**
	Northern Africa	82 x 10^5^	12 x 10^3^**(0.14)**	39 x 10^3^**(0.48)**	96 x 10^3^**(1.2)**
	Total	29 x 10^6^	22 x 10^5^**(7.6)**	33 x 10^5^**(11.3)**	46 x 10^5^**(15.7)**
Domestic	Western Africa	60 x 10^5^	26 x 10^4^**(4.3)**	47 x 10^4^**(7.9)**	81 x 10^4^**(13.4)**
	Central Africa	66 x 10^5^	28 x 10^4^**(4.2)**	50 x 10^4^**(7.7)**	80 x 10^4^**(12.2)**
	Southern Africa	27 x 10^5^	88 x 10^3^**(3.3)**	18 x 10^4^**(6.8)**	37 x 10^4^**(14.0)**
	Eastern Africa	58 x 10^5^	11 x 10^4^**(1.9)**	30 x 10^4^**(5.2)**	71 x 10^4^**(12.3)**
	Northern Africa	82 x 10^5^	64 x 10^2^**(0.08)**	42 x 10^3^**(0.51)**	20 x 10^4^**(2.4)**
	Total	29 x 10^6^	74 x 10^4^**(2.5)**	15 x 10^5^**(5.1)**	30 x 10^5^**(10.0)**

The southern Democratic Republic of Congo (DRC), Burundi, Rwanda, eastern Zambia, Malawi, Tanzania, parts of Kenya, Uganda, Zimbabwe, Mozambique and the north-east of South Africa all appear to have areas of relatively high potential suitability for repeated introduction of ASF to domestic pigs from sylvatic cycles.

#### Objective 2

**Figure 3:**
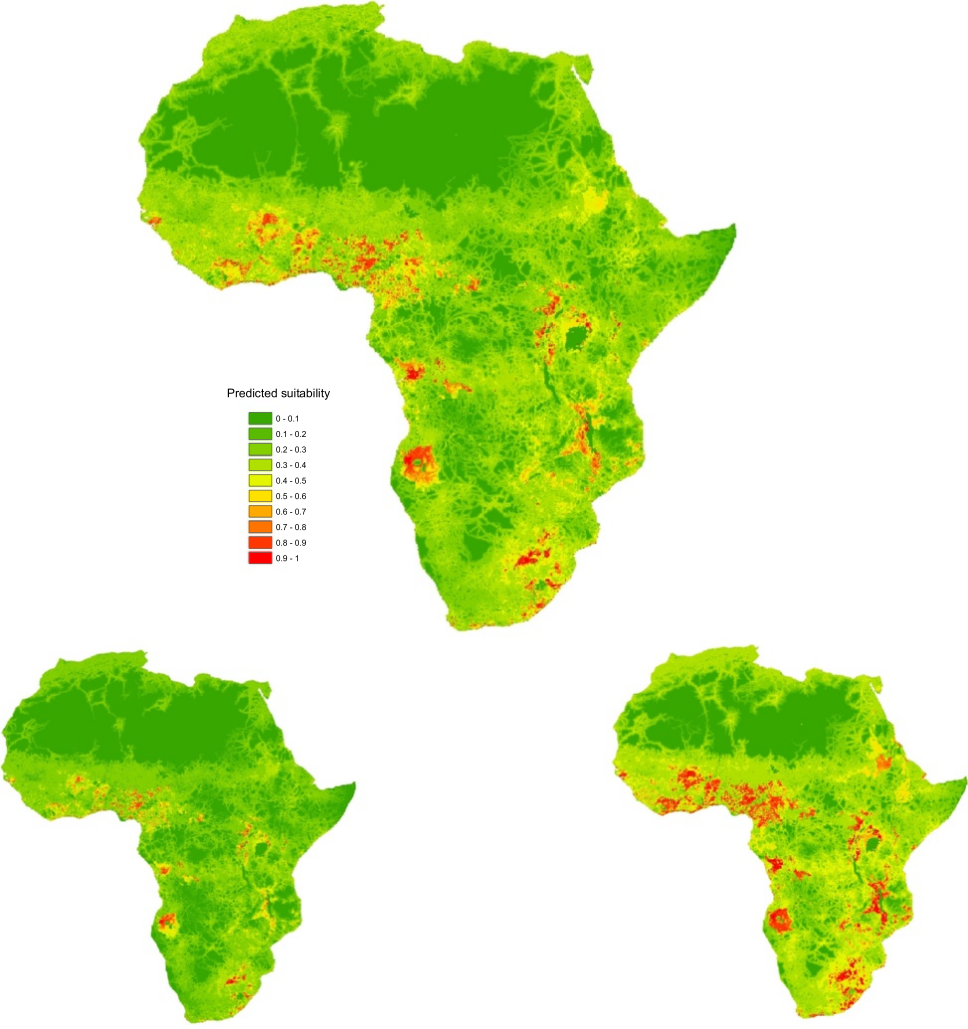
**Suitability for ASF persistence as part of domestic cycles (5**^**th **^**percentile (left); average (middle); 95**^**th **^**percentile (right)).**

Areas with apparently high suitability for cycles involving sustained spread of ASF within domestic pig population pathways can be found in all regions of Africa excluding Northern Africa (Figure 3 and Table 7). Countries with the highest suitability for sustained spread within domestic pig populations were predicted to be in South Africa, Malawi, Nigeria, Angola, Cameroon, Burkina Faso, Benin, Togo, Guinea-Bissau, Rwanda and Burundi, as well as smaller parts of Uganda and the DRC.

### Validation

**Figure 4:**
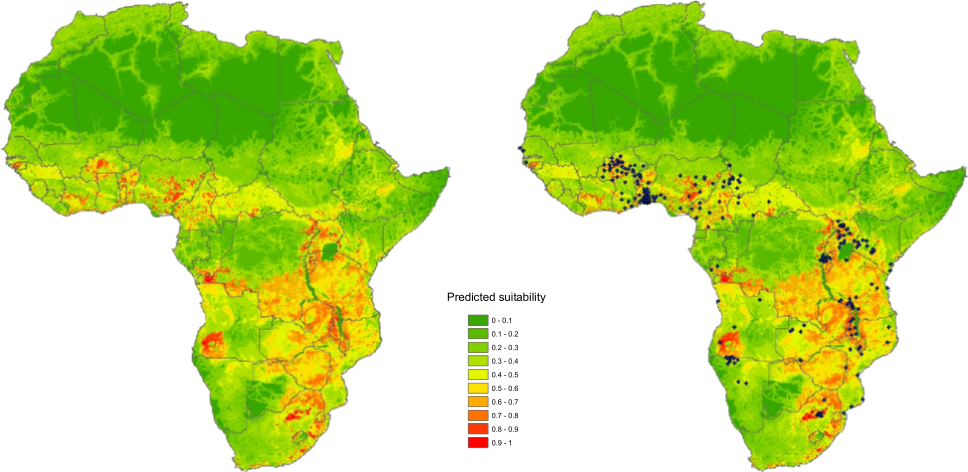
Combined estimates of mean suitability for ASF endemicity in domestic pig populations, with location of ASF outbreaks between 2005 and 2012 overlaid (right).

A total of 252 primary ASF outbreak reports from 20 countries were identified. The combined suitability estimates showed a very high degree of visual agreement with the outbreaks reported to the OIE (Figure 4). The average partial ROC AUC ratio for the combined sylvatic and domestic suitability estimate was 1.28 (range 1.20 – 1.36), where 0 of 1000 iterations resulted in a ROC AUC ratio ≤1, suggesting a consistently good degree of model predictability for the reported ASF outbreaks. The average AUC ratio when considering mean domestic suitability alone was 1.46 (range 1.30 – 1.62) and was 1.08 (range 1.01 – 1.14) for sylvatic cycles.

## Discussion

To our knowledge, this is the first attempt to predict the spatial distribution of ASF in Africa using a standardised approach. The combined suitability estimates derived from the MCDA procedure reveal a good degree of agreement with the distribution of cases reported to the OIE between 2005 and 2012, although the number of officially reported ASF cases are likely to represent only a small proportion of those that actually occur across Africa, particularly in areas in which the disease is endemic. The predicted distribution of areas with high suitability for repeated introduction of ASF from wildlife reservoirs and for sustained transmission within pig populations also supports the anecdotal distribution of sylvatic and domestic pig cycles identified by a number of authors [1,2,5,44].

Outputs from the MCDA procedure suggest that most of western, south-western, and west-central Africa are relatively unsuitable for repeated introduction of ASF from wildlife reservoirs. African swine fever is endemic or approaching a situation of endemicity in several countries in these areas, including Nigeria [45], Senegal [31], Guinea Bissau [46], and Angola [47]. There are areas of widespread suitability for domestic ASF transmission in these countries, and it is likely that the persistence of ASF in such areas is the result of sustained domestic pig transmission cycles, without the need for repeated introduction from wildlife reservoirs [5,20].

Countries that are predicted to have widespread suitability for repeated introduction from sylvatic reservoirs are Tanzania, Zimbabwe, and Malawi. The southern DRC, eastern parts of Zambia, and smaller areas of Uganda, Kenya, Angola, Botswana, South Africa, Burundi, Rwanda and Mozambique also appear to be highly suitable for ASF transmission associated with sylvatic cycles. Despite this apparently high suitability, the majority of recent ASF outbreaks in these regions are likely to have been caused by the movement of pigs and their products rather than sylvatic spill over [48-50]. We also predict that all of these countries, except Botswana, have a widespread distribution of areas of apparent high suitability for sustained transmission as part of domestic pig cycles. This is particularly the case in Angola, Malawi, Uganda, South Africa and the western DRC. The potential importance of transmission as part of domestic rather than sylvatic cycles in these areas, and continental Africa as a whole, is supported by the considerably higher predictive ability (based on average AUC ratio) of domestic suitability estimates for all areas in which ASF outbreaks have been reported compared to the combined suitability estimates and those for sylvatic cycles alone. However, sporadic introduction from wildlife reservoirs should not be ignored as potential risk in such areas [44,51]. Indeed, although bushpigs were given a moderate weight in this study, recent work implicated *P. larvatus* as a possible source of ASF infection for domestic pigs living in close proximity to a National park in Kenya [52]. African swine fever viruses isolated from warthogs living on a cattle ranch in central Kenya were also shown to be genetically similar to those causing recent outbreaks in Kenya and Uganda [53]. Hence, it is quite possible that sporadic introduction of ASFV from sylvatic reservoirs could lead to onward transmission via domestic cycles, particularly in those areas of Central, East and Southern Africa where we predict high suitability for both transmission routes.

Through the use of a stochastic approach, this study has attempted to incorporate uncertainty into several of the key decision making steps in the MCDA procedure, and particularly into the assignment of relative importance scores to risk factor layers. The BetaPert distribution has been widely used for modelling expert opinion [54], and allowed us to incorporate the uncertainty that existed in our subjective assessment of the available evidence. To our knowledge, this is the first example of the formal introduction of stochasticity into a spatial MCDA in the area of disease or health mapping. Such a step is particularly important in the knowledge-driven mapping of diseases such as ASF where, in the absence of quantitative epidemiological studies, the contribution of several putative risk factors is unclear [14,16] and, as in this study, a highly subjective approach is used to assess this contribution. The overall geographic range of suitability for repeated transmission of ASFV as part of the domestic or sylvatic cycle was generally the same across the distribution of estimates. However, the application of a somewhat arbitrary (but commonly used [41]) threshold of 0.5 to dichotomise the output into unsuitable and suitable reveals the extent and impact of our uncertainty on the percentage coverage of areas of potential suitability for either cycle.

Pairwise-comparison is a method that has been applied widely to assess the importance of one factor over another in a decision-making process [38,55], and has been used previously for disease mapping using spatial MCDM [16,17]. An important issue in the pairwise-comparison procedure is that matrices are often inconsistent, potentially leading to senseless decision making [56]. Through the use of an iterative approach, in which the pairwise-comparison matrix is populated by values drawn at random from a distribution defining each pairwise-comparison, it is likely that the frequency of inconsistency in the construction of the matrix and resulting estimation of factor weights was increased in this study. Although the rule described by Saaty [37] was used to combat such inconsistency, the validity of the 10 % cut-off in the resultant consistency ratio has been questioned [57]. Kwiesielewicz and Uden [58] showed that a matrix that passes a consistency test successfully may still be contradictory. Hence, it is acknowledged that, on the basis of the stochastic approach adopted, some of the allocated weights selected from the distributional ranges used may have been the result of inconsistent comparisons. However, given that the resultant risk maps are the summarised estimates of a large number of independent weight combinations, the impact of any such inconsistency is likely to be small.

It is important that outputs from knowledge-driven risk mapping are interpreted in the light of the methodology adopted, which remains highly subjective and combines data sources of variable quality, most of which originated from internet-based data repositories. Moreover, the approach can only incorporate those risk factors that can be mapped. Trade of pigs and their products is likely to be a major mechanism by which ASFV spreads within and between regions [1]. The movement of middle-men between farms, for example, has been reported as an important risk factor in the spread of ASFV in Africa [8], but such activities cannot be mapped directly in the absence of spatial data describing such movements. Proximity to major market centres was used as a proxy for this, and other such trade-related activities, but the use of such a variable for this purpose cannot be expected to capture the local scale variability in the movement of animals and contaminated people, equipment and products that may contribute to virus spread.

The MCDA approach relies on knowledge that already exists: to date, there have been few quantitative epidemiological studies of ASF in Africa, and whilst MCDA provides a useful framework with which to aggregate the work that has been conducted, it remains limited by the scope of the available evidence. Given the limited consensus on risk factors for ASF in the literature, our model used limited number of criteria: as additional studies on the epidemiology of ASF in Africa are conducted, the criteria to describe ASF risk and in particular the perceived relative importance of these criteria, could be expected to change [20].

Moreover, this study is an interpretation of the existing knowledge and relationships between these risk factors by the authors only, since no other experts were consulted. Whilst we attempted to limit the potential impact of our subjective assessments through modelling the relative importance of criterion using probability distributions, a different set of experts may have chosen different minimum, most likely and maximum values for the BetaBert distributions, and thus derived somewhat different suitability estimates. The predictions from this work should be should be interpreted accordingly, but it would be reasonable to expect, given the current state of knowledge on ASF in Africa, that another set of experts’ average suitability estimates using the same set of criteria would be likely to fall between the 5^th^ and 95^th^ percentiles of our own.

## Conclusion

This study used a limited number of known or hypothesised risk factors for ASF and our own subjective assessment of the importance of those risk factors to predict the distribution of ASF persistence via sylvatic and domestic transmission cycles in Africa. The suitability predictions derived are not intended to be interpreted as definitive, instead, they provide a ‘best guess’ estimate based on the currently available evidence, and our own interpretation of that evidence. With these limitations in mind, outputs from this study indicate that areas of high suitability for transmission as part of domestic cycles occur throughout sub-Saharan Africa. Areas of sylvatic suitability are restricted to Central, East and Southern Africa, but commonly overlap areas of suitability for domestic cycles. Hence, whilst domestic transmission of ASF is likely to be the major means by which the disease persists throughout sub-Saharan Africa, and should probably continue to be the focus of disease control and prevention efforts, the continued potential for spill over from wildlife reservoirs should not be ignored.

## Abbreviations

AMD: African Mammals Databank; AUC: Area under the curve; ASF: African swine fever; ASFV: African swine fever virus; CI: Consistency index; CR: Consistency ratio; DRC: Democratic Republic of Congo; GFH: Giant Forest Hog; GIS: Geographic information system; MCDA: Multi-criteria decision analysis; OIE: World Organisation for Animal Health; ROC: Receiver operating characteristic; WAHID: World Animal Health Information Database; WLC: Weighted linear combination.

## Competing interests

No author has any competing interest to declare.

## Authors’ contributions

WAdeG, SC, BW and DUP designed the study. WAdeG performed data analysis as part of the study. All authors contributed to the development of risk factor weightings. All authors made contributions to the conception, design, and revision of the manuscript. All authors read and approved the final manuscript.
